# Lung Recurrence of Papillary Thyroid Cancer Diagnosed With Antithyroglobulin Antibodies After 10 Years From Initial Treatment

**DOI:** 10.3389/fendo.2018.00590

**Published:** 2018-10-09

**Authors:** David Viola, Laura Agate, Eleonora Molinaro, Valeria Bottici, Loredana Lorusso, Francesco Latrofa, Liborio Torregrossa, Laura Boldrini, Teresa Ramone, Paolo Vitti, Rossella Elisei

**Affiliations:** ^1^Unit of Endocrinology, Department of Clinical and Experimental Medicine, University of Pisa, Pisa, Italy; ^2^Unit of Pathological Anatomy, Department of Surgical, Medical, and Molecular Pathology and Critical Care Medicine, University of Pisa, Pisa, Italy

**Keywords:** thyroid cancer (TC), TgAb, recurrence, BRAF mutation, lymphocytic thyroiditis

## Abstract

**Introduction:** Papillary thyroid cancer (PTC) is the most common endocrine malignancy. More than 98% of patients achieve an excellent response with no evidence of clinical, biochemical, or structural disease after initial treatment. In these patients structural recurrence is rare, more frequently diagnosed in the first 5 years from initial treatment and almost invariably localized in neck lymph nodes.

**Patient:** We report the case of a woman affected by PTC who presented with rapidly rising anti-thyroglobulin antibodies (TgAb) level after 10 years from clinical, morphological and biochemical remission.

**Diagnosis and Treatment:** In 2003, a 56 year old patient was treated with total thyroidectomy and radioiodine remnant ablation (RRA) for a PTC (2 cm) with minimal extrathyroidal extension (T3N1aM0 according to the 6th AJCC TNM staging system) associated with diffuse lymphocytic thyroiditis. In 2004 the patient was free of disease defined as undetectable Tg after recombinant human TSH administration in the absence of TgAb and structural disease. Since February 2012 the appearance and progressive increase of TgAb titer was observed and in 2014 a ^18^FDG-PET scan documented three hypermetabolic lesions suggestive of lung micrometastases. The lung lesions were cytologically confirmed as PTC metastases. Both the primary tissue and the lung metastasis were positive for BRAF *V600E* mutation. The patient was treated with 131-radioiodine that showed radioiodine avid lung lesions that lose the ability to take up iodine at the following treatment. The patient is still alive and the lung lesions are growing slowly.

**Conclusions:** Structural recurrence in patients that demonstrated an excellent response after initial treatment for PTC is extremely rare, and distant metastases exceptional but possible. This case is peculiar because recurrence was early identified after 10 years from initial treatment for the presence of detectable TgAb in a patient that had an histological diagnosis of lymphocytic thyroiditis but with an atypical clinical presentation (normal thyroid at neck ultrasound and undetectable TgAb and anti-thyroid peroxidase antibodies). For this reason TgAb should be tested with Tg in patients with a history of lymphocytic thyroiditis, either histological or humoral, also when TgAb is in the normal range and not suggestive of autoimmune thyroiditis.

## Background

Thyroid carcinoma (TC) is the most common endocrine malignancy accounting for about 4% of all human tumors (1). The most frequent histotype is PTC accounting for 85–90% of all TC, followed by follicular thyroid cancer (FTC), medullary thyroid cancer (MTC), poorly differentiated thyroid cancer (PDTC), and anaplastic thyroid cancer (ATC) representing 5–10, 3–5, 2–3, and 1% of all TC, respectively (2). Well-differentiated thyroid carcinoma (DTC), both PTC and FTC, originate from follicular cells and maintain the ability to concentrate iodine and producing Tg. These features of differentiation have important clinical and prognostic implications. In fact, the slow growth rate of differentiated PTC and the possibility to treat it with radioactive iodine make it one of the most curable human cancers (3). Despite an overall survival of 98.1% after 5 years from diagnosis PTC recurrence rate is rather high reaching in some series 30% (4, 5). In the vast majority of cases, structural recurrent disease is represented by neck lymph nodes and can be suspected on the basis of detectable levels of serum Tg and easily diagnosed with neck ultrasound followed by fine needle aspiration biopsy. Although more frequent in the first 5 years after initial treatment, recurrent disease could manifest also after 10–20 years (4).

Although Tg is a quite well-recognized and reliable tumor marker, in subjects with detectable/high titer of anti-thyroglobulin antibodies (TgAb), that can interfere in its measurement, Tg is no longer trustable as tumoral marker (6, 7). In these cases TgAb titer can be used as a surrogate marker since its disappearance is correlated with the clinical remission of the disease while its persistence or increase can suggest the persistence or recurrence of the disease (8–10). In fact, after the initial treatment (i.e., total thyroidectomy and RRA) thyroid autoantibodies, specifically TgAb, decrease over the following years disappearing in a mean of 3 years when the disease is cured (11). At variance, the persistence or increase of TgAb titer should induce the suspicious of a persistent or recurrent disease and further diagnostic tests should be performed (12). On this regard, several studies have shown that changes in the TgAb levels can predict the risk of persistence/recurrence in TgAb-positive PTC patients while patients who obtain undetectable TgAb titer have an excellent prognosis (13). More recently, the evidence that an increase in TgAb titer should not be undervalued was described also in subjects that were not treated with radioiodine and could indicate the presence of either normal or tumoral thyroid tissue (14). However, while the previous reported series were focused on patients with PTC and positive TgAb from the beginning, the present case is peculiar because the recurrence was discovered for the appearance, followed by a progressive increase, of TgAb titer that was negative at the time of PTC diagnosis. To our knowledge no similar cases have been reported so far.

## Case presentation

**Figure 1:**
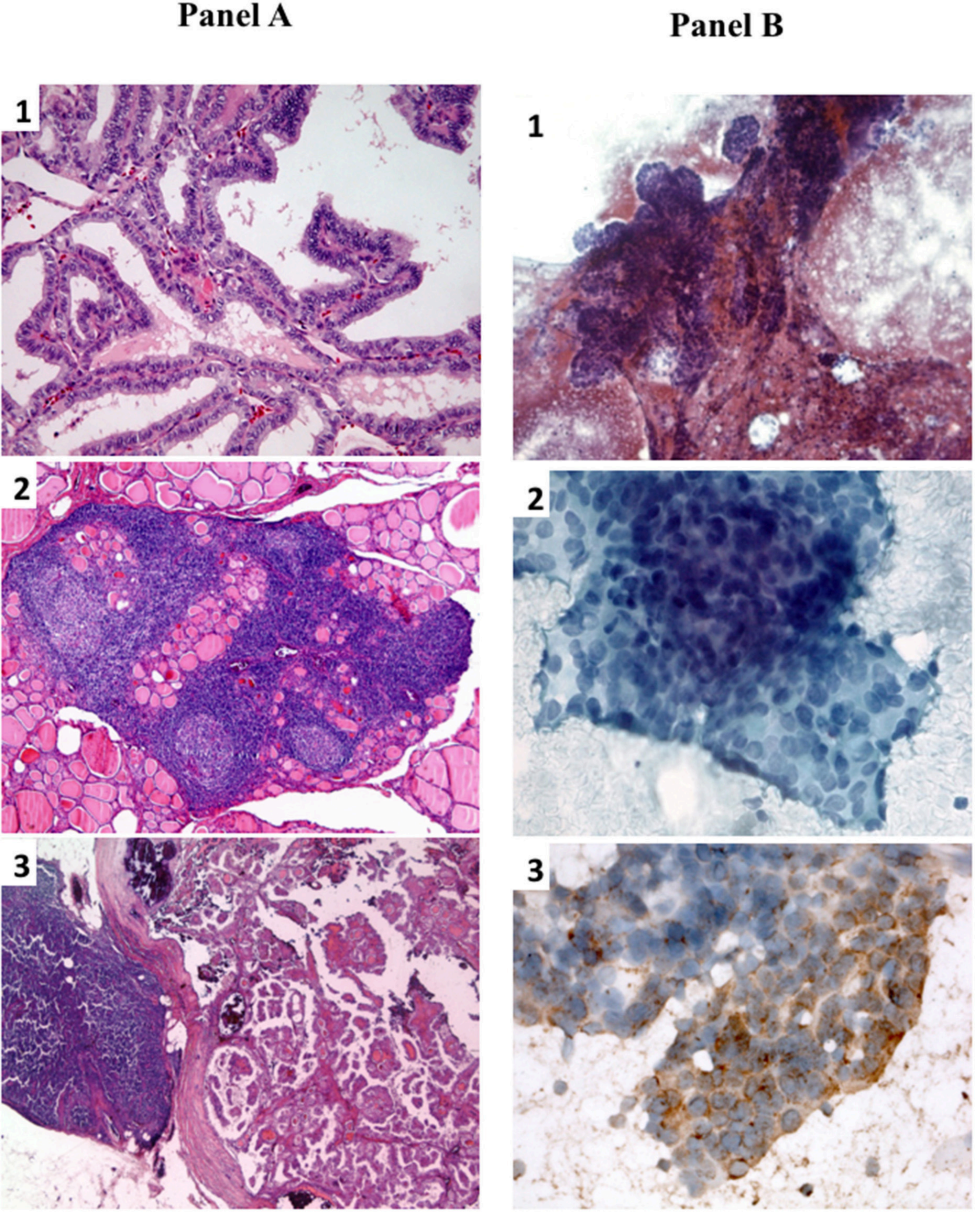
**(A)** Histological slides of primary PTC and lymph node metastasis. **(A1)** Primary PTC with well-formed papillary structures (20X, hematoxilyn/eosin); **(A2)** collateral diffuse lymphocytic thyroiditis with lymphoid follicles and germinal centers(5X, hematoxilyn/eosin); **(A3)** lymph node metastatic lesion of PTC (2.5X, hematoxilyn/eosin). **(B)** Fine-needle aspiration cytological smears of lung metastastic lesion. **(B1)** low power magnification showing papillary-like structures with smooth contours and palisading of nuclei (10X, papanicolau stain); **(B2)** high power magnification showing nuclear characteristics of PTC (finely granular chromatin and one pseudoinclusion) (40X, papanicolau stain); **(B3)** immunocytochemistry showing cytoplasmatic positivity for thyroglobulin, confirming the thyroid origin of the lung lesion (40X).

On January 2003 a 56-year-old woman underwent total thyroidectomy for a multinodular goiter with a thyroid nodule that was suspicious for malignancy at cytology. No evidences of biochemical and/or ultrasonographic features of autoimmune thyroiditis were present before surgery (15). The histological diagnosis was papillary thyroid carcinoma (PTC), classical variant (Figure 1A1) but with focal areas of tall cells, perithyroid soft tissue invasion, and multifocality. Histology showed also the presence of a diffuse lymphocytic infiltration (Figure 1A2).A few central compartment lymph node metastases were also present (Figure 1A3) (pT3mN1aMx according to the 6th AJCC-TNM staging system) (16).

On May 2003 the patient was referred to the Endocrine Oncology Unit of the Department of Clinical and Experimental Medicine of the University Hospital of Pisa to perform radioiodine remnant ablation (RRA) with 30 mCi of 131-I, after levothyroxine (L-T4) withdrawal. Post-therapeutic whole body scan (pWBS) showed an exclusive uptake in the central neck that was suggestive for thyroid remnant, serum thyroglobulin (Tg) was 1.2 ng/ml with undetectable levels of TgAb. On May 2004 the patient had undetectable Tg (i.e., <0.5 ng/ml) serum (Immulite 2000 Thyroglobulin; DPC, Los Angeles, CA) after the administration of recombinant human thyroid stimulating hormone (rhTSH; Thyrogen; Sanofi Genzyme, Cambridge, Massachusetts), negative TgAb (AIA-Pack 2000, Tosoh Corporation, Tokyo, Japan) and negative neck ultrasound (US). Considering the excellent response to the initial treatment the patient, accordingly to the American Thyroid Association guidelines (17), was considered in clinical remission and then followed with clinical and biochemical (i.e., Tg and TgAb) controls and neck US every 12–24 months. The clinical evaluations, neck US and both Tg and TgAb were negative and/or undetectable for the following 5 years.

**Figure 2:**
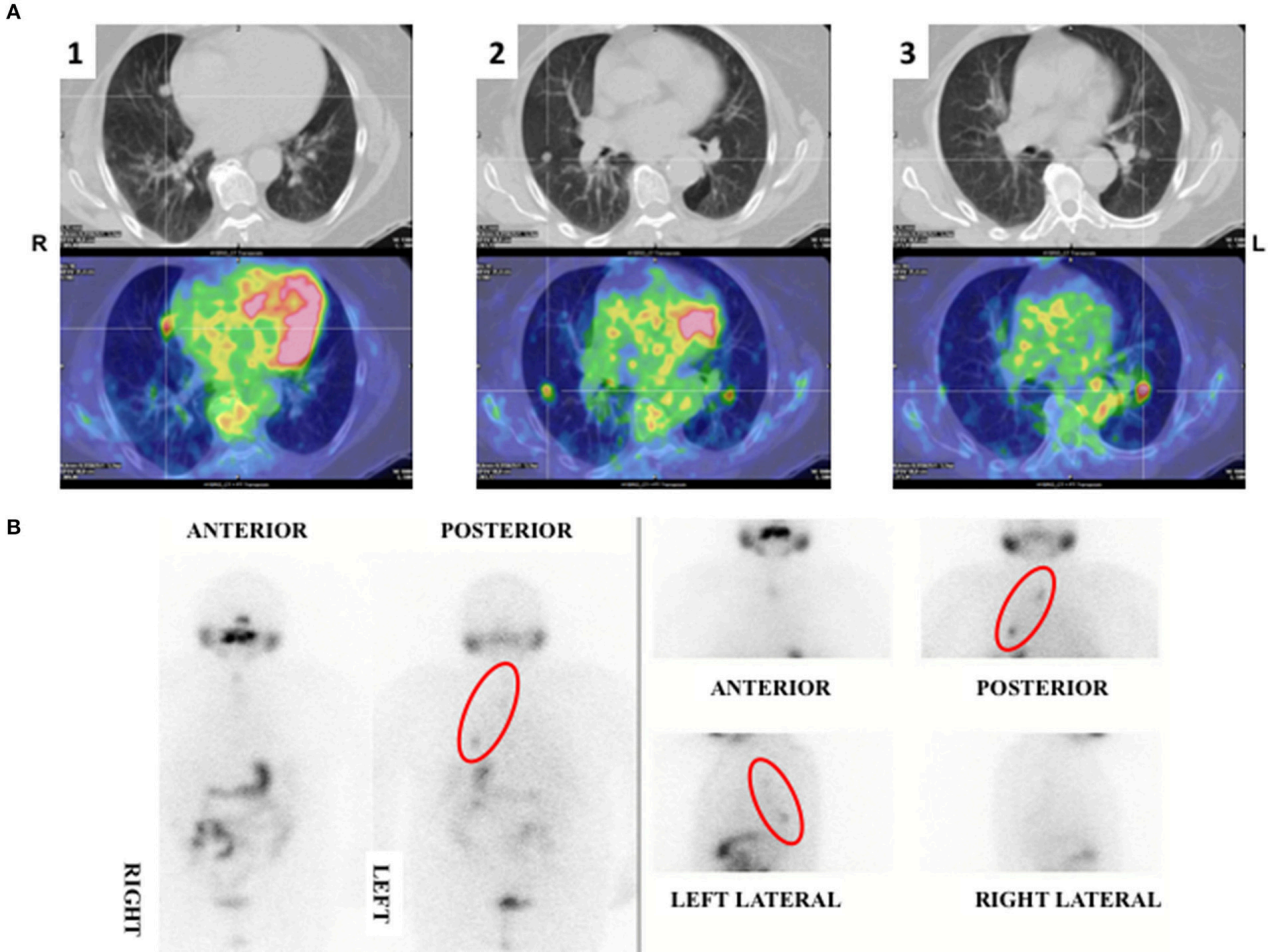
**(A)** ^18^FDG-PET scan showing three hypermetabolic nodular lung lesions localized at the medial (1) (SUV max 3.1) and lateral (2) (SUV max 2.7) segment of the right medium lobe and at the apical segment (3) of the left inferior (SUV max 3.9) lobe of the lungs. **(B)** pWBS showing 131-I uptake corresponding to the hypermetabolic lung lesions localized at the medial segment of the right lobe and at the left inferior lobe.

**Figure 3:**
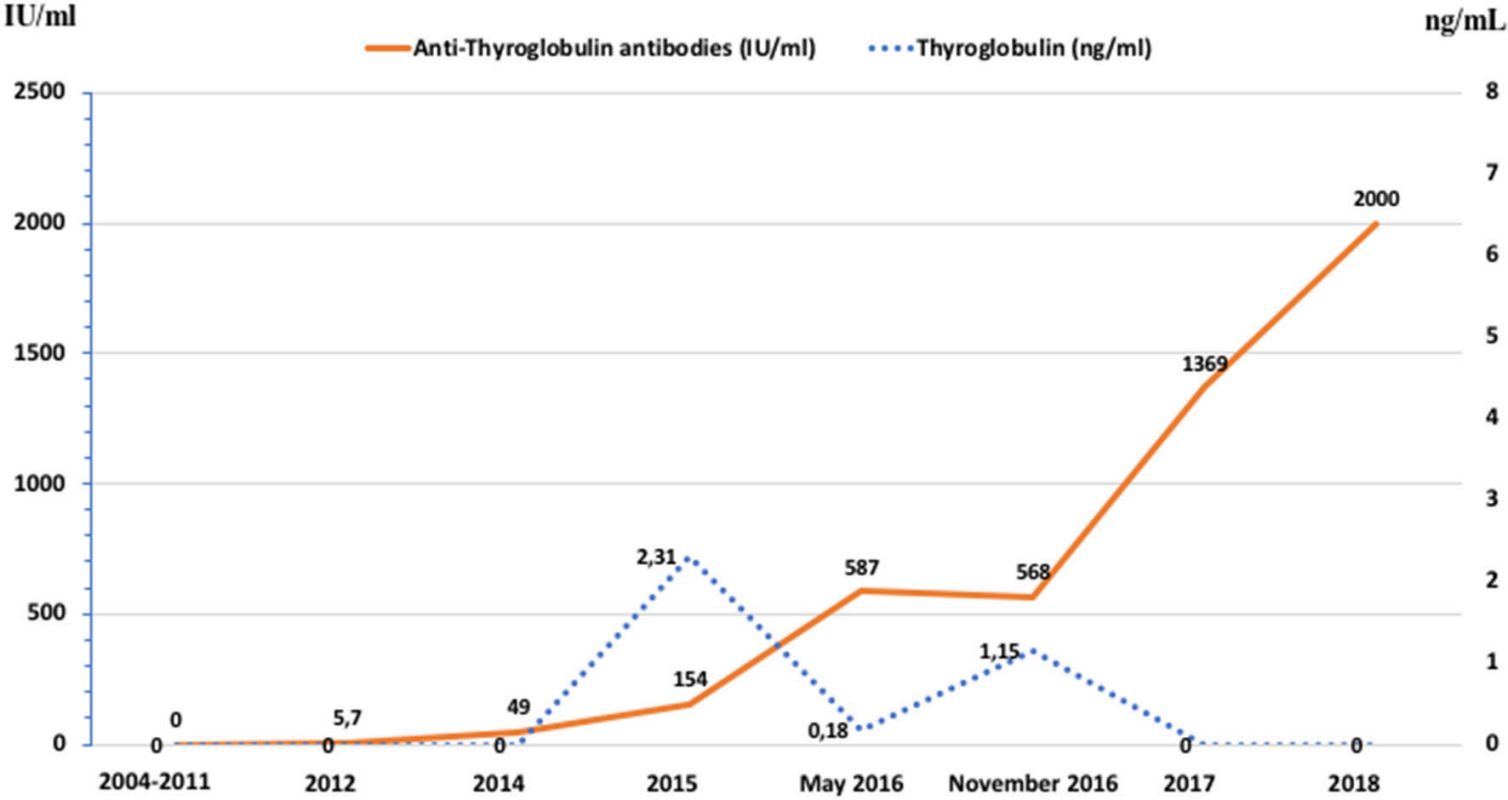
Graphic representation of the patient's serum Tg values and TgAb titer between 2004 and 2018.

In 2012 an unexpected positive serum TgAb titer was noted still in the absence of detectable serum Tg. A small (8 mm) indeterminate lymph node was newly detected at neck US. The titer of TgAb slightly increased over the years and for this reason on August 2015 the patient was subjected to a computerized tomography (CT) scan that showed three small lesions (maximum diameter 12 mm) in the lung. A ^18^Fluorodeoxyglucose-Positron Emission Tomography (^18^FDG-PET) scan confirmed the presence of these lesions that were hypermetabolic (Figure 2A). On November 2015 the largest lung nodule, that was located in the inferior left lobe, was subjected to fine needle biopsy and cytology confirmed that cell morphology was suggestive of PTC (Figures 1B1,2). Moreover, the immunohistochemistry was positive for TTF-1 and focally for Tg (Figure 1B3) and the measurement of Tg in the wash out of the needle used for the lung cytology was 1780 ng/ml, confirming the thyroid origin of the lesion. On December 2015 the serum Tg became slightly detectable and the patient was treated with 150 mCi of 131-I. The pWBS showed two areas of uptake in the lung that were suggestive of iodine avid lung metastases and likely corresponding to two of the lesions found at the CT and PET scan (Figure 2B).On May 2016 serum biomarkers, namely TgAb, continued to increase and the patient was subjected to a CT scan that showed a slight increase of the lung lesions (maximum diameter 14 mm). Taking into account the slow but continue increase of serum biomarkers on December 2016 the patient was treated with additional 150 mCi of 131-I. At that time the pWBS didn't show any radioiodine uptake. On February 2018 the CT scan showed a further increase in one lung lesion that reached a maximum diameter of 22 mm. A detailed history of serum biomarkers (i.e., Tg and TgAb) variation is summarized and shown in Table 1 and Figure 3.

**Table 1:** Patient's serum Tg values and TgAb titer between 2004 and 2018.

**Date**	**Tg (ng/mL)**	**TgAb (IU/mL)**
2004–2011	0	0
Feb-2012^*^	0	5.7
Nov-2014	0	49
Dec-2015	2.31	154
May-2016	0.18	587
Nov-2016	1.15	568
Oct-2017	0	1,369
Feb-2018	0	2,000

* *Since 2012 Tg measurement was performed with an ultrasensitive method (Beckman Coulter, Inc., Fullerton CA) with a functional sensitivity of 0.1 ng/mL while TgAb assay used was the same during all the follow-up period*.

Paraffin embedded slices of the primary tumor tissue and cytological smears of the metastatic lung lesion were used for the DNA extraction that was targeted sequenced with a next generation sequencing system (Ion S5 deep sequencer, Ion Torrent, Applied Biosystem) by using a custom panel designed to analyze all thyroid related oncogene mutations. The analysis showed the presence of BRAF *V600E* mutation with an allelic frequency of 18 and 27% in the lung and primary tumor tissue, respectively. No other alterations were found with this analysis.

According to the Hospital rules, the patient signed an informed consent for the use of her clinical data and biological specimens for research purposes and publication of this case report; the study was approved by the Internal Review Board.

## Discussion

Papillary thyroid carcinoma is a lymphotropic tumor, especially in young patients, which shows a high lymphatic spread at diagnosis while distant metastases are quite rare and account for <5% of all TC patients, including other histotype (5). The most frequent sites of PTC metastatization, excluding lymph nodes and in order of frequency, are lungs, bone, and liver. Although the presence of a persistent disease after initial treatment, either biochemical and/or structural, has been reported up to 20–30% in different series of DTC, structural recurrent disease (i.e., the reappearance of disease after a period of documented cure) is not so frequent and it is present in about 1–2% of cases (18, 19). Disease recurrence rate depends mainly on tumor histology, gender, patient's age, and stage of the disease at diagnosis. This risk is very well-predicted by the risk of recurrence categories (i.e., low, intermediate and high) described in the American Thyroid Association guidelines (17). The periodical monitoring of serum Tg allows to perform an early diagnosis and the cure of these rare cases. Although the first 5–10 years after the initial treatment are those with the highest rate of recurrence, a lifelong follow-up is suggested because of the late onset recurrences (4).

In about 20–25% of PTC an autoimmune thyroiditis with circulating TgAb and/or TPOAb, is associated to the tumor (20). In these cases, the follow-up requires the concurrent measurement of Tg and TgAb because of the interference of the latter on the Tg assays and in many cases TgAb acquire the role of “Tg surrogate” marker (21, 22). However, although cases of PTC with a lymphocytic infiltration in the absence of concomitant serum autoimmunity are also described (21), no studies on the need to measure TgAb also during the follow-up of these patients have been reported so far and our case, although peculiar, testifies this need.

About 60% of PTC are positive for BRAF *V600E* mutation and its prognostic role for recurrence and/or mortality is still under debate (19, 23–26). Nevertheless, there are evidences that BRAF *V600E* positive cases have a lower degree of differentiation and, as a consequence, a lower ability to take up iodine (23, 27, 28). It is worth to note that this case was harboring a BRAF *V600E* mutation with a rather high allelic prevalence both in the primary and in the lung metastases. This finding can at least partially support the hypothesis that this mutation could be the driver of the recurrence and the cause of low uptake and response to radioiodine (19).

In conclusion, the description of this case is relevant not only because the recurrence with distant metastases in a PTC patient who achieved excellent response is exceptional but also because the way in which recurrent disease was diagnosed is unusual. In particular, this case underlines the importance to always measure serum TgAb along with Tg in TC patients not only for the interference that the antibodies can determine on Tg measurement but also for their role as Tg surrogate marker. The case demonstrates that this is particularly relevant when histological lymphocytic infiltration is present even if serum TgAb are negative. In fact the appearance of the positivity of TgAb allowed the early diagnosis of disease recurrence and the appropriate treatment and follow-up the patient.

## Ethics statement

The study was approved by the patient and Internal Review Board.

## Author contributions

RE obtained funding, DV and RE study concept, design and drafting of the manuscript, FL critical revision of the manuscript. DV, RE, LA, EM, VB, LL, FL, PV, and RE acquisition and interpretation of data, LT, LB, and TR technical and/or material support.

### Conflict of interest statement

The authors declare that the research was conducted in the absence of any commercial or financial relationships that could be construed as a potential conflict of interest.
